# Modelling COVID-19 Scenarios for the States and Federal Territories of Malaysia

**DOI:** 10.21315/mjms2021.28.5.1

**Published:** 2021-10-26

**Authors:** Noor Atinah Ahmad, Mohd Hafiz Mohd, Kamarul Imran Musa, Jafri Malin Abdullah, Nurul Ashikin Othman

**Affiliations:** 1 School of Mathematical Sciences, Universiti Sains Malaysia, Pulau Pinang, Malaysia; 2 School of Medical Sciences, Universiti Sains Malaysia, Kubang Kerian, Kelantan, Malaysia

**Keywords:** singular spectrum analysis, transition dynamics, data-driven approach, COVID-19 forecast

## Abstract

Severe acute respiratory syndrome coronavirus 2 (SARS-CoV-2) causes COVID-19 disease, which has become pandemic since December 2019. In the recent months, among five countries in the Southeast Asia, Malaysia has the highest per-capita daily new cases and daily new deaths. A mathematical modelling approach using a Singular Spectrum Analysis (SSA) technique was used to generate data-driven 30-days ahead forecasts for the number of daily cases in the states and federal territories in Malaysia at four consecutive time points between 27 July 2021 and 26 August 2021. Each forecast was produced using SSA prediction model of the current major trend at each time point. The objective is to understand the transition dynamics of COVID-19 in each state by analysing the direction of change of the major trends during the period of study. The states and federal territories in Malaysia were grouped in four categories based on the nature of the transition. Overall, it was found that the COVID-19 spread has progressed unevenly across states and federal territories. Major regions like Selangor, Kuala Lumpur, Putrajaya and Negeri Sembilan were in Group 3 (fast decrease in infectivity) and Labuan was in Group 4 (possible eradication of infectivity). Other states e.g. Pulau Pinang, Sabah, Sarawak, Kelantan and Johor were categorised in Group 1 (very high infectivity levels) with Perak, Kedah, Pahang, Terengganu and Melaka were classified in Group 2 (high infectivity levels). It is also cautioned that SSA provides a promising avenue for forecasting the transition dynamics of COVID-19; however, the reliability of this technique depends on the availability of good quality data.

## Introduction

The new coronavirus called severe acute respiratory syndrome coronavirus 2 (SARS-CoV-2) (or previously known as 2019-nCov) and the disease associated with this virus, COVID-19, have been discovered in December 2019 (1). COVID-19 has spread across the globe and became a major life-threatening pandemic. This coronavirus also causes severe global health problems in many countries like USA, India, Brazil, Malaysia and Italy, with significant number of death tolls can be seen around the world (2–3). The number of the confirmed cases worldwide was 217,926,107 with a total death of 4,524,102 people, as of 30 August 2021 (4). In general, the coronavirus has spread rapidly throughout different continents, with around 221 countries and territories having reported the presence of COVID-19 infected cases (5).

**Figure 1:**
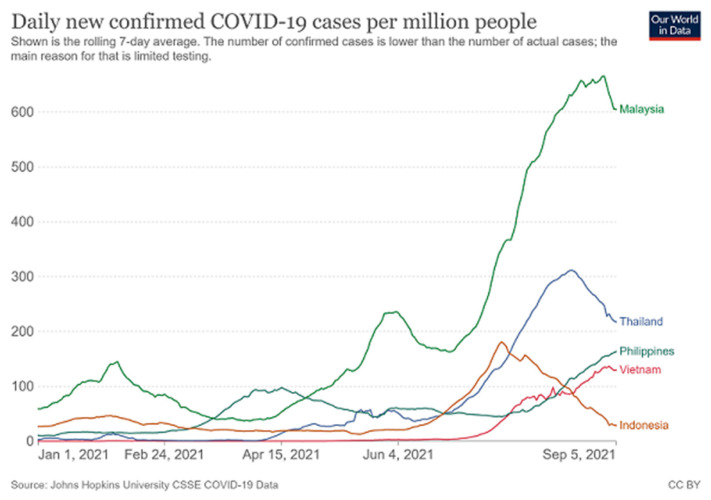
Daily new confirmed COVID-19 cases per million people for Malaysia, Thailand, Philippines, Vietnam and Indonesia (1 January 2021–5 September 2021). Source: (6)

**Figure 2:**
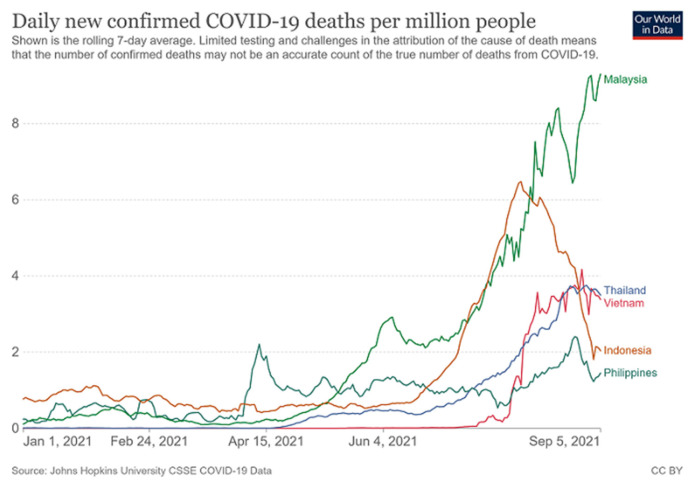
Daily new confirmed COVID-19 deaths per million people for Malaysia, Thailand, Vietnam, Indonesia and Philippines (1 January 2021–5 September 2021). Source: (6)

As of 30 August 2021, Malaysia had recorded 1,725,357 confirmed cases with 16,382 deaths and 265,713 active cases (4). This unfortunate situation has placed a great burden on the healthcare systems and put many people in Malaysia at risk (3, 7). The trend of daily new confirmed COVID-19 cases and COVID-19 deaths per million people from 1 January 2021 until 5 September 2021 for Malaysia, Thailand, Vietnam, Indonesia and Philippines are shown in Figures 1 and 2, respectively. Malaysia has remained the country with the highest percapita daily new cases and daily new deaths between the five countries. Currently, the healthcare systems in some states in Malaysia for e.g. Pulau Pinang, Johor, Sabah and Sarawak seem unable to cope with these extremely difficult circumstances with serious number of new infections daily (3, 8). While Selangor (in particular, Klang Valley) appears to contribute to the highest daily new cases in Malaysia, there has been some noticeable indications that the daily active cases in this state are decreasing and the infection dynamics have also demonstrated a sign of easing off.

To better understand this infectious disease spread in Malaysia and obtain an insight on the infectivity levels in distinct states and federal territories, we employ a mathematical modelling approach using a Singular Spectrum Analysis (SSA) technique (9) to forecast COVID-19 transmission. The main modelling objective is to understand and classify the transition dynamics of COVID-19 in Malaysia (instead of focussing on point forecasting). Generally, SSA is a linear approach to the analysis and prediction of time series, and this technique is widely used in various fields such as finance, engineering, medicine and biology. Recently, SSA approach has also been used by scientific communities to predict COVID-19 spread in several countries such as Saudi Arabia and Malaysia (10–11). The salient features of SSA are that this technique is non-parametric, and it is also a data-driven approach, which does not rely on any prior modelling assumptions (11). Due to the promising potential of this modelling framework to predict future trend, we reckon that SSA can provide a reliable forecast for COVID-19 spread in different regions in Malaysia.

## Data Analysis Using Singular Spectrum Analysis

In this study, we analysed 16 time series of the number of daily cases (Malaysia plus 15 states) from 25 January 2020 to 26 August 2021. Four 30-days ahead forecasts are produced for each time series using the major trend from singular spectrum analysis. Each major trend describes > 50% of the total variance of the respective data. Description of each forecast is given in Table 1.

## Results and Trend Analysis

Figures 4 and 5 depict a graphical view of the forecasts for the number of daily cases for Malaysia as well as the forecasts for each state. We analyse the trends according to the transient behaviour of the forecast as it changes from A to D. The inclination of each forecast indicates the level of infectivity, i.e. if the forecasts become steeper as they change from A to D, it gives an indication that the infectivity is still intense. On the other hand, if the forecasts become flatter, it gives an indication that infectivity is easing off. The detailed analysis is given in Table 2.

Based on the analysis in Table 2, we have divided COVID-19 situation in Malaysia into four categories:

Category 1: Infectivity is high with the potential of getting worse.
Category 2: Although numbers are still on the increase, there are signs that the infectivity begins to get under control. We can afford to be cautiously optimistic.
Category 3: Infectivity decreases fast which signals a greater control of the spread of the virus. We can be optimistic that the number of cases will continue to reduce.
Category 4: Infectivity has decreased to a level where the disease can be eradicated.

## Discussion

**Figure 3:**
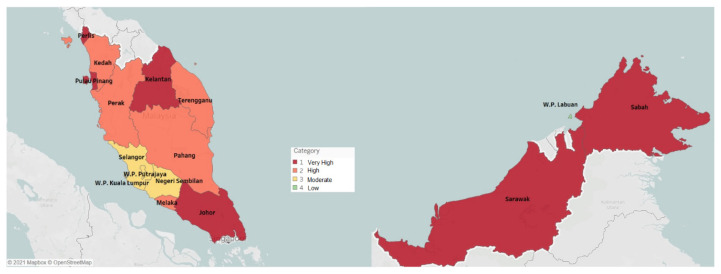
Hotspot mapping predicted by the SSA technique, which is used to forecast COVID-19 spread for the states and federal territories in Malaysia

**Figure 4:**
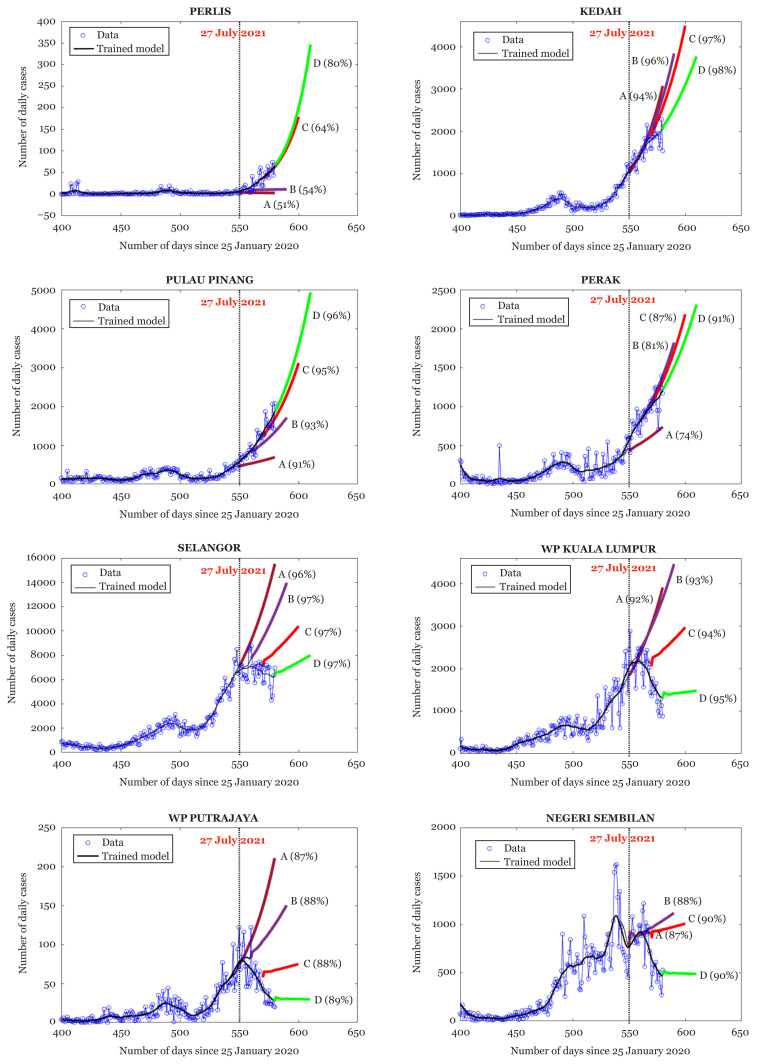
30-days ahead forecasts for Perlis, Kedah, Pulau Pinang, Perak, Selangor, Federal Territory of Kuala Lumpur, Federal Territory of Putrajaya and Negeri Sembilan. The value in the round bracket next to each forecast gives the percentage of total variance described by the trend from which the respective forecast is generated

**Figure 5:**
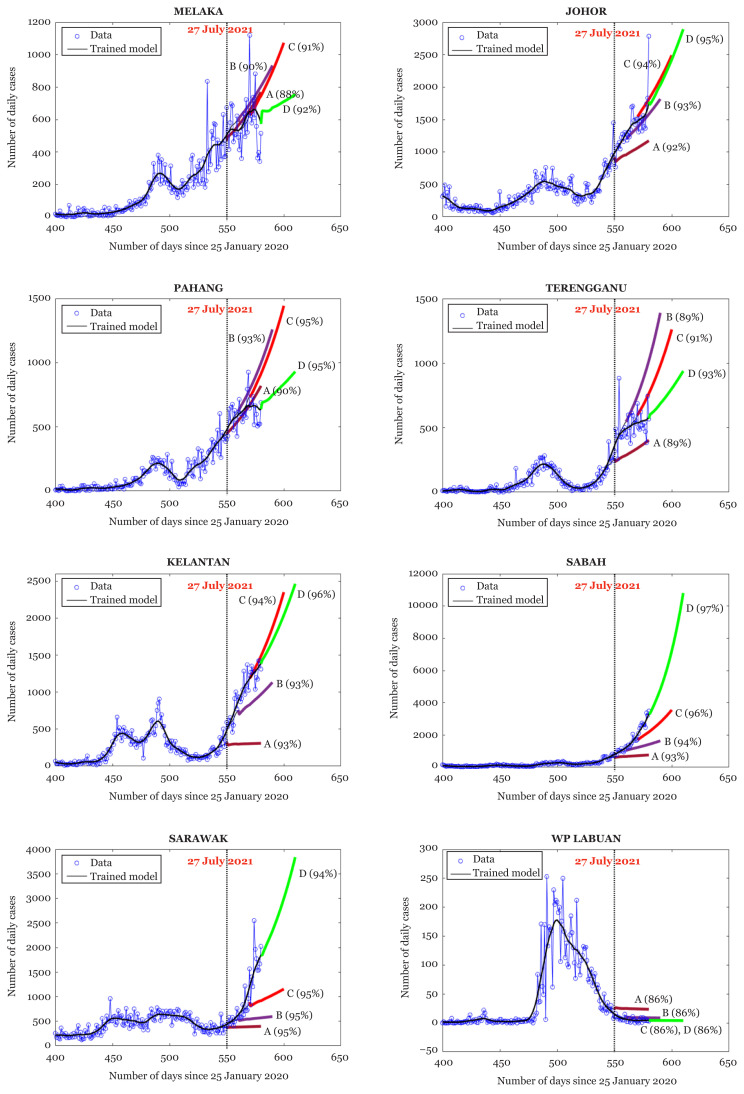
30-days ahead forecasts for Melaka, Johor, Pahang, Terengganu, Kelantan, Sabah, Sarawak and Federal Territory of Labuan. The value in the round bracket next to each forecast gives the percentage of total variance described by the trend from which the respective forecast is generated

Based on the modelling analysis in Table 2 (and Figures 4 and 5), we summarise the classification of Categories 1, 2, 3 and 4 using hotspot mapping as shown in Figure 3. Overall, the COVID-19 spread has progressed unevenly across states and federal territories. The highest infectivity levels have been forecasted to occur in Pulau Pinang, Sabah, Sarawak, Kelantan and Johor. These transient forecasts are also in parallel with the statistics produced by Ministry of Health Malaysia where some of these states (e.g. Pulau Pinang, Sabah and Sarawak) portray the trend of highest incidence (per 100,000 people) of COVID-19 cases in Malaysia. One of reasons that contributes to this trend is due to a significant increase in COVID-19 Delta variant cases in several states like Sarawak and Pulau Pinang. Other states like Johor, Sabah, and Kelantan also face resurgence in COVID-19 infections and deaths due to rather low vaccination rates in Malaysia.

The smallest state in Malaysia, Perlis, has also seen an increase in the daily COVID-19 cases over the past few months, which can be attributed to the non-compliance to SOP and the opening of the economic sectors under Phase Three of the National Recovery Plan. Based on SSA analysis, other states like Perak, Kedah, Pahang, Terengganu and Melaka are classified as high infectivity levels; however, it is believed that the spread begins to get under control given that the imposition of distinct pharmaceutical (e.g. vaccination, treatment) and non-pharmaceutical (e.g. wearing of face mask, social distancing, quarantine) intervention strategies is being implemented strictly.

While the most densely populated region in Malaysia, Klang Valley (which consists of capital city, Kuala Lumpur, and its most populous state, Selangor) has the highest number of cases (in terms of daily new infections), we realise that the infectivity levels demonstrated a rapid decreasing trend, which signals a greater control of the COVID-19 spread in these regions. Klang Valley, Putrajaya and Negeri Sembilan have also recorded a turnaround in infection trend following a ramp-up in vaccinations, with more than 80% of its adult population is fully vaccinated. Only one federal territory, i.e. Labuan, has shown a low infectivity level and the spread of COVID-19 disease can be eradicated in this region. We believe the main contributing factor is the high vaccination rate resulting in lower community transmission and lower hospital admissions; this situation could be seen with the results from the Operation Surge Capacity in the Klang Valley and states such as Negeri Sembilan and Labuan where large majority of their populations have been fully vaccinated.

## Conclusion

Overall, we have demonstrated the applicability of SSA techniques in the analysis of COVID-19, particularly for distinct states and federal territories in Malaysia. Just like any other data-driven methods, the quality of forecasts from SSA can only be as reliable as the data itself. The capability of SSA to reveal unknown trends is quite evident, but good quality data is important to realise its true potential in predicting the transmission dynamics of COVID-19. As the country faces the inimitable challenges of COVID-19 head-on, the health care capacities in Malaysia can be seen to be continuously overwhelmed. To mitigate the pressure on our health systems, numerous pharmaceutical and non-pharmaceutical intervention measures need to be imposed so as to save lives and minimise the virus’ spread. It is hoped that this work would motivate modellers as a call to arms and decision makers as a guide for forecasting outbreaks, planning of resource allocation, and making informed decisions to control the spread of this pandemic effectively.

## Figures and Tables

**Table 1:** 30-days ahead forecasts produced for each dataset

Forecast	Description
A	Training data: 25 January 2020–27 July 2021 Forecasts: 28 July 2021–26 August 2021
B	Training data: 25 January 2020–06 August 2021 Forecasts: 7 August 2021–5 September 2021
C	Training data: 25 January 2020–16 August 2021 Forecasts: 17 August 2021–15 September 2021
D	Training data: 25 January 2020–26 August 2021 Forecasts: 27 August 2021–25 September 2021

**Table 2:** Analysis of trends

Data	Description	Infectivity easing off?	Category
Perlis	Forecasts become steeper as they change from A to D	No	1
Kedah	All the forecasts are still quite steep, however, they get flatter as they change from A to D	Begins to ease off	2
Pulau Pinang	Forecasts become steeper as they change from A to D	No	1
Perak	Forecasts B, C, and D are still quite steep, however, they get flatter as they change from B to D	Begins to ease off	2
Selangor	Forecasts become flatter persistently as they change from A to D	Yes	3
Federal Territory of Kuala Lumpur	Forecasts become flatter persistently as they change from A to D	Yes	3
Federal Territory of Putrajaya	Forecasts become flatter persistently as they change from A to D	Yes	3
Negeri Sembilan	Forecasts become flatter persistently as they change from A to D	Yes	3
Melaka	Forecast D (most recent) begins to flatten	Begins to ease off	2
Johor	Forecasts become steeper as they change from A to D	No	1
Pahang	Forecast D (most recent) begins to flatten	Begins to ease off	2
Terengganu	Forecasts become flatter slowly as they change from A to D	Begins to ease off	2
Kelantan	Forecasts become steeper as they change from A to D	No	1
Sabah	Forecasts become steeper as they change from A to D	No	1
Sarawak	Forecasts become steeper as they change from A to D	No	1
Federal Territory of Labuan	Infectivity appears to be under control. All forecasts appear to flatten almost completely.	Yes	4
